# LabTrove: A Lightweight, Web Based, Laboratory “Blog” as a Route towards a Marked Up Record of Work in a Bioscience Research Laboratory

**DOI:** 10.1371/journal.pone.0067460

**Published:** 2013-07-23

**Authors:** Andrew J. Milsted, Jennifer R. Hale, Jeremy G. Frey, Cameron Neylon

**Affiliations:** 1 Department of Chemistry, University of Southampton, Southampton, United Kingdom; 2 ISIS Neutron Facility, Science and Technology Facilities Council Rutherford Appleton Laboratory, Harwell Science and Innovation Campus, Didcot, United Kingdom; University of Illinois-Chicago, United States of America

## Abstract

**Background:**

The electronic laboratory notebook (ELN) has the potential to replace the paper notebook with a marked-up digital record that can be searched and shared. However, it is a challenge to achieve these benefits without losing the usability and flexibility of traditional paper notebooks. We investigate a blog-based platform that addresses the issues associated with the development of a flexible system for recording scientific research.

**Methodology/Principal Findings:**

We chose a blog-based approach with the journal characteristics of traditional notebooks in mind, recognizing the potential for linking together procedures, materials, samples, observations, data, and analysis reports. We implemented the LabTrove blog system as a server process written in PHP, using a MySQL database to persist posts and other research objects. We incorporated a metadata framework that is both extensible and flexible while promoting consistency and structure where appropriate. Our experience thus far is that LabTrove is capable of providing a successful electronic laboratory recording system.

**Conclusions/Significance:**

LabTrove implements a one-item one-post system, which enables us to uniquely identify each element of the research record, such as data, samples, and protocols. This unique association between a post and a research element affords advantages for monitoring the use of materials and samples and for inspecting research processes. The combination of the one-item one-post system, consistent metadata, and full-text search provides us with a much more effective record than a paper notebook. The LabTrove approach provides a route towards reconciling the tensions and challenges that lie ahead in working towards the long-term goals for ELNs. LabTrove, an electronic laboratory notebook (ELN) system from the Smart Research Framework, based on a blog-type framework with full access control, facilitates the scientific experimental recording requirements for reproducibility, reuse, repurposing, and redeployment.

## Introduction

The traditional laboratory notebook is the cornerstone of the scientific record [1]. A researcher is expected to record all the detail necessary to enable another researcher, who is assumed implicitly be knowledgeable, to replicate the work [2] [3]. To quote from Day [4]

*“Faraday's hand-written notebooks…have long been of interest to historians and philosophers of science because of the extraordinarily direct insight they give into the way his thinking developed…. They are also remarkable in the amount of detail that they give about the design and setting up of experiments, interspersed with comments about their outcome and thoughts of a more philosophical kind. All are couched in plain language, with many vivid phrases of delightful spontaneity….”*

Leaving aside for the moment the question of how often this ideal is realised in practice, the format of the traditional paper laboratory notebook neither enables nor facilitates the sharing and discussion of protocols recorded, the safe storage of data that is not readily captured in paper form, or, for most modern instruments, even the actual recording of the raw data. Some recent high media profile exchanges have highlighted some of the issues of showing data and – importantly - methods. The teaching mantra of “show your workings” [5] is well worth remembering at all stages of a research career. These issues make an appealing case for the Electronic Laboratory Notebook (ELN).

### Electronic Laboratory Notebooks (ELN)

ELNs are available in a wide range of implementations, from research prototypes to industrial-strength commercial systems. In their simplest forms, ELNs provide an electronic analogue of paper, supplemented by data storage facilities. While such basic systems are straightforward and do enable text-based searching, they do not exploit the full potential of a computer system to systematise and catalogue data. At the opposite extreme is an implementation of a fully semantically aware product, for example the Semantic Electronic Laboratory Notebook work at the University of Southampton. The *Smart Tea* project [6] [7] [8] [9] [10] aimed both to guide a synthetic organic chemist through a synthesis and to produce a fully semantically annotated record of what had occurred captured directly as an RDF graph (Resource Description Framework, W3C, Available from: http://www.w3.org/RDF/, the data description component of the Semantic Web [11] [12] [13] [14]).

The metadata framework for the *Smart Tea* process was based on the assessment form used for the Control of Substances Hazardous to Health (COSHH), a health and safety requirement related to the handling of potentially hazardous materials. COSHH is the UK legislation that requires a full safety audit to be undertaken prior to using hazardous materials so as to minimize or mitigate the risks involved. The practical outcome of this is that prior to undertaking an experimental investigation a full description of the materials, the procedures, the likely outcomes and potential hazards and how these would be dealt with if they occur needs to be written down on an institutionally approved COSHH form and appropriately signed off.

The *Smart Tea* architecture extended the framework to include an RDF representation of the experimental plan, which was interpreted to provide prompts to the chemist with a place provided for adding experimental details and observations (i.e. metadata in advance). The result was a series of RDF statements (triples) that described the procedures undertaken and acted as a provenance chain for the materials produced. The *Smart Tea* approach worked well using a tablet PC in the laboratory, and the project has been continued via the *More Tea* work [15], which provides a richer set of semantics that more accurately reflect the nature of the work of the synthetic chemist. More recently a deeper look at the semantics of experiment planning and enactment led to the “Planning and Enactment Ontology” formally described by its project title as the oreChem ontology [16].

In the current work we have taken an alternative approach that uses the familiar framework of a WebLog (Blog) to combine in a pragmatic way the provenance capture of the semantically aware systems with the journal characteristics of the traditional notebook for use as a collaborative laboratory notebook.

### From semantically aware notebooks to blogs

Our experience with *Smart Tea* found that methodology could be too heavyweight and prescriptive and, potentially, significant work would be needed to adapt the approach to other domains of experimental science. We therefore decided to investigate a Web 2.0 approach involving the addition of minimal semantics to otherwise unstructured data, rather than going for a full semantic web system. We also wanted to develop the notebook from the perspective of the individual researcher, and to avoid being constrained by the requirements of specific subjects, such as chemistry or biology.

The blog is a component of science communication familiar to, and popular with, a growing number of researchers [17] (ScienceBlogs available from: http://scienceblogs.com/). Blog systems allow almost complete freedom in what is recorded but also enable the organisation and categorisation of data in a machine-readable format. Moreover, the use of blogs would be familiar to incoming graduate students, so we adopted the idea of a laboratory blog as an ELN, and developed the software that would eventually evolve into the LabTrove system. In this paper we describe the use of a researcher-centric blog as the ELN for a bioscience research project.

### Blogs as ELNs: limitations of blogging systems

A blog naturally provides the basis for a web-based laboratory recording system, but at the outset of this project in 2006 the standard blog engines lacked many of the features required to generate a genuinely useful laboratory notebook tool. A key problem was the access to and security of a complete revision record. In blog engines, date stamping and maintaining records of how pages have changed is generally limited, whereas wikis place a strong emphasis on these areas. Some groups have elected to use wikis in preference to blogs for this reason; alternatively a blogging engine could be modified to incorporate versioning.

One of the major benefits identified in ELN deployments in industry is the reduction in the time required for recording repeated procedures. Most users would adopt a copy-and-paste approach, but such techniques are prone to error. To enable easy-to-use and efficient recording of repeated laboratory procedures, template structures, as provided with most office systems, are required. If supported by purpose-built tools, such templates would save valuable time and help to promote the consistent description of related processes. Moreover, templates provide a means of embedding semantic metadata.

Support for metadata is limited in most blog systems. WordPress (http://wordpress.org/) and many other web platforms offer simple tagging for categorising posts. Laboratory work and processes can sometimes require more hierarchical systems to categorise projects and sub-projects, and classes of procedure, data, or material [18]. However, such structures do not always adapt well to the somewhat haphazard and contingent processes of experimental research. Finding a balance that usefully describes the rich information context of the research process, while retaining sufficient flexibility to respond rapidly to day-to-day events in the laboratory is a major socio-technical challenge, and one that is not well met by standard blog frameworks.

Representing objects that are not located on the web leads to a conceptual difficulty when using a blog framework to capture the important events, data, and materials that feature in a research process. With the naïve application of a blog as a simple journal this issue does not arise: physical objects can be referenced in the page by names, numbers, or characteristics. However, if we wish to exploit the power of the web as a network [19], we must consider how to manage and represent those *physical* objects in that network, especially if we want to deploy the tools of graph analysis and search, which depend on the rich information provided by links between *digital* objects.

### Blogs as ELNs: support for linked laboratory data

The promise of linked data [20], the web of objects (e.g. chemical biology applications [21]) and the emerging (although currently incomplete) range of semantic web tools [22] provide a powerful motivation to exploit the potential of a web-based laboratory record [11] [23]. There is particular scope for providing links between digital records and physical samples and artefacts. Neylon has discussed what a ‘web-native’ laboratory record would look like, emphasising the potential of unique identifiers i.e. Uniform Resource Identifiers (URIs) for research elements, notification mechanisms that provide annotated lists of recently created objects, and tools that facilitate the creation of links between those objects [24].

The ‘nouns’ of research, the data and physical samples, can then be linked together by ‘verbs’, the physical and digital processes involved in experiments and analysis. In turn a catenation or chain of these processes, providing links between what we have used and what we have created as a result, represents a close analogue of the traditional laboratory notebook. A web-based laboratory notebook can embody a network of relationships between all research elements, a network that can be analysed effectively by both the traditional graph-based algorithms of online search engines and, if properly annotated, by the emerging tools of the linked-data web. In principle, this linking methodology provides a lightweight and user-friendly route towards a fully semantically annotated research record.

This methodology requires three components: unique identifiers in the form of URIs; sets of newly created objects; and sufficient metadata for each object to make the research record machine-readable. The object sets therefore need to be provided in a form that is sufficiently structured to enable machine-based parsing and categorisation. The object sets must also be in a form that can be manipulated by tools that can easily create links to and from the newly created objects.

Blogs offer an appealing framework for realizing the linking methodology. We can represent the research objects as posts, which automatically generate permalinks that become the URIs for those objects. For notification, all blog frameworks generate RSS (www.w3schools.com/rss/) or ATOM feeds (“What is Atom?” AtomEnabled available from: http://www.atomenabled.org/), which also provide annotation metadata, including date and time stamps, author identifiers, and URIs.

The main limitation to realizing the linking methodology lies in the weak metadata support provided by conventional blog frameworks. Free-text tagging, while extensible and highly flexible, is plagued by inconsistent usage, leading inevitably to multiple tags for the same subject, often differing minimally in spelling, and the lack of common understanding of the semantics of tags. On the other hand, structured and controlled vocabularies can be too rigid for users, particularly those working out of the initial scope for which such systems were designed. There are places where well-defined schemes with strong guidance are absolutely appropriate, such as in defined programs of biomedical research where a large number of parallel assays are carried out on clearly defined samples that form part of a defined research investigation. Where these formalisms do not map as well to the mental models and practice of the research process imposing strong requirements can lead to poorer or parallel recording practices.

In this paper we describe the development of an ELN that exploits the linking support inherent in blog-based platforms and augments the authoring facilities with an extensible and flexible metadata framework.

## Initial Explorations of the Laboratory Blog Framework: Identifying System Requirements

Our aim was to explore a blog-based approach to recording laboratory processes and objects. We investigated the potential of a number of blogging engines that are widely used and identified issues that limited their direct application in our context. In particular, we identified the following needs: support for recording of repeated processes; improved support for versioning; and metadata richer than that provided by simple tagging systems.

We based our original framework on the open source μblog framework, which is no longer accessible but can be found on the web archive [25]. We supplemented its customary blog functionality with a key-value pair system that enabled a more sophisticated categorisation of posts than that provided by simple tags. Initially, we also imposed the restriction that a published post could not be changed. In this section we describe our initial use of this blogging system as a laboratory notebook, then known as the LaBLog system, and illustrate some of the issues that guided the ultimate design and use of our current system, LabTrove (LabTrove. Available from: http://www.labtrove.org/documentation/).

### The blog as a simple journal notebook

The journal is a natural starting model when using a blog as a laboratory notebook system, effectively creating a web-based analogue of the paper notebook. The digital nature of a blog provides advantages over paper, including automated backup of data and protocols, and the ability to do simple text searches. The comment facility, common to most blogs, allows notes to be taken during the course of an experiment and for other researchers to comment, ask questions, or offer advice.

**Figure 1:**
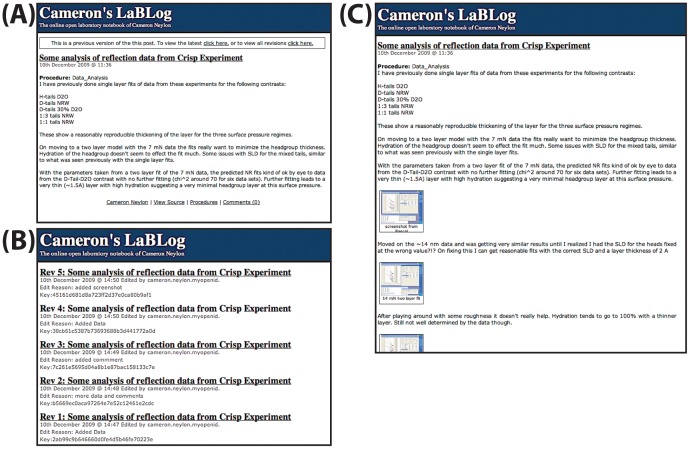
This sequence illustrates how posts develop as a process is recorded or minor mistakes are corrected. The LabTrove system archives all versions of the post and they are accessible from the final version of the post. All the URLs resolve to a open science laboratory notebook and should be visible. (A) The first version of a post describing an analysis procedure (http://biolab.isis.rl.ac.uk/camerons_labblog/11928/Some_analysis_of_reflection_data_from_Crisp_Experiment.html?revision=11928 Accessed 6 March 2013). (B) The record of revisions, showing the first five revisions of the post (http://biolab.isis.rl.ac.uk/camerons_labblog/11928/Some_analysis_of_reflection_data_from_Crisp_Experiment.html?revisions, Accessed 6 March 2013). (C) The final version of the post including further analysis and images (http://biolab.isis.rl.ac.uk/camerons_labblog/11928/Some_analysis_of_reflection_data_from_Crisp_Experiment.html?revision=11938, Accessed 6 March 2013). Copies of these web pages are provided as part of the supplementary material as well as the links to the Labtrove instance at ISIS.

Early in the project we encountered problems with the need to edit posts, mainly to correct typographical and other errors but also for adding observations over the course of extended experiments. This difficulty was identified by other groups, and prompted the move from blog to wiki at UsefulChem [26]. Our initial assumption that the record as first recorded should remain unchanged quickly proved unworkable. Modifications or errors in the record can be noted as comments but this separates this information from the core of the post and makes it more difficult to read and parse the record overall. In many cases it is also desirable to keep notes as an experiment proceeds, saving the details on a regular basis. Moreover, with an immutable digital record there is no immediately accessible analogue of simply crossing out a journal page and rewriting it. To surmount this issue, we developed a simple revision versioning system that enables posts to be edited and changed or supplemented, but requires a reason for each edit and makes the full record of changes accessible (see Figure 1).

### The nature of a post

Having chosen to use a blog we have taken the view implicitly that the laboratory notebook will comprise a series of individual (and interlinked) posts. This raises the very important question: what is, or should be, the appropriate content, or indeed size, of a single post? Our initial answer was “one experiment”. However, it became evident that it was not necessarily clear what “one experiment” actually meant!

The first use of our blog system as a simple journal can be seen in the early entries (Nov/Dec 06) on the Beta-Glu blog (available from http://blogs.chem.soton.ac.uk/beta_glu). We recorded experimental procedures either in free text or in tables; data, generally images, were uploaded to the post that recorded the experimental procedure. A post could cover all stages from the preparation of samples, through their processing to their analysis. In some cases it was unclear which analysis related to which sample: there was no informational link between a specific procedure, its input materials, and its outputs or analysis. While our prototype enabled a laboratory record to be captured, its approach did not readily support more sophisticated analysis or categorisation of the elements of the record.

By focussing on when it was desirable to identify a specific instance of a sample, we resolved one aspect of the informational link problem: we adopted the policy of a single post for each physical sample and each data file. Problems frequently arise in experimental research where knowledge of the precise identity of a sample used is useful (“exactly which sample did you use, was it the same one as was used over here?”) and this approach helped to reduce these issues by uniquely identifying samples and data files.

**Figure 2:**
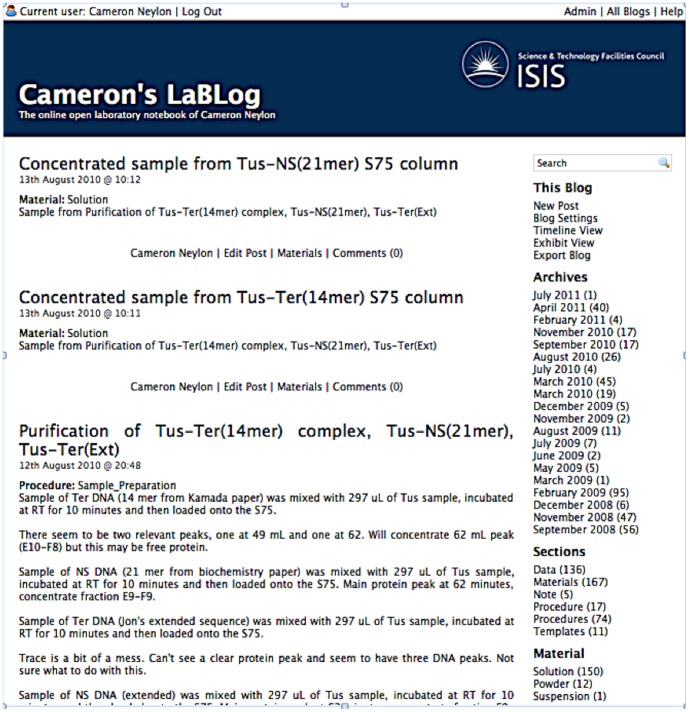
Extract from a laboratory notebook recorded as a blog, presented in a standard format, and showing three individual posts. The top two posts represent samples whereas the bottom post is a procedure. This view is accessible at http://biolab.isis.rl.ac.uk/camerons_labblog/month/1280617200, Accessed 6 March 2013.

Adopting the *one-item one-post* system also enabled us to represent the relationships between research elements with hyperlinks between the relevant posts (see Figure 2). This system also allowed us to use the key-value metadata to capture the characteristics of individual posts rather than using metadata to describe the relationships between posts. Using the metadata in this way led us to adopt broad categories of post: ‘material’, ‘procedure’, ‘data’, which are used reasonably consistently alongside personal sub-categories that often differ from user to user. This broad consistency makes it straightforward to apply a range of tools generically across a set of laboratory notebooks. Consistency is not enforced anywhere in the system, but consistent practices allow the additional tools to work better.

### Conclusions from the initial investigation: specific requirements

The two important conclusions from our initial investigation were that our organizational approach arose naturally from our need efficiently to record and present the data; and the identification of a specific set of technical and interface requirements as development targets. The system must enable and support the publishing and linking of posts that refer to individual research elements: samples, procedures, and datasets.

The use of a blog-based system, and the ability to link posts together, naturally led us to use those links to describe relationships: other approaches using metadata did not work. As we applied critical analysis to how we were recording our work, the system itself was encouraging us to develop and redevelop our approach. This iterative methodology based on our actual use of the system enabled us to develop the blogging software system now known as LabTrove (http://www.labtrove.org/) the implementation of which we will now describe in more detail.

## Implementation of the LabTrove System

We implemented the LabTrove blog system as a server process written in PHP (http://www.php.net/), using a MySQL database (http://www.mysql.com/) to persist posts and other objects. Details of the PHP implementation are provided in the System Description (The LabTrove System Description is provided as File S1), which incorporates specific sections about the server process and the database. The source code and documentation are available as an open source project via SourceForge (http://sourceforge.net/), the version used for the later part of the work reported here is available at http://sourceforge.net/projects/labtrove/files/Old%20Versions/2.2/labtrove-2.2-r358.tar.gz/download). LabTrove is also available for installation from the project website: www.labtrove.org, which also provides full documentation.

In this section we discuss the functionality of LabTrove and how it is used in practice. We cover the principles and implications of the *one-item one-post* system; we describe the use of templates to support repeated procedures; and outline how LabTrove facilitates the publication of laboratory records. We illustrate these features with examples from a bioscience application from the laboratory of one of the authors (CN).

### LabTrove in practice

The LabTrove user interface adopts a standard blog format, although we now refer to the blogs themselves as e-Notebooks, thus distinguishing the blog content from its implementation. By default the posts are displayed in reverse chronological order with a right hand navigation bar that provides related information, such as links to categories of content and posts partitioned by publication date. The navigation bar also provides alternative viewing options for the e-Notebook, including a timeline view of all posts, and an ‘Export Blog’ option. Individual posts can be exported as XML or as a PNG image. The interface also provides a search box. For a fuller description of the user interface, refer to the relevant section in the System Description. LabTrove displays each post with the following meta-information: author, date of publication, and categorisation, together with options to view or (if the user is the author of the post) edit the post and to add a comment to the post.

### Publishing a post

Having logged in, the user clicks ‘New Post’ in the navigation bar, which opens a new page with text boxes for the title and body of the post. Posts are marked up with BBcode (Bulletin Board Code, http://bbcode.org), a language choice inherited from the original μBlog framework. A toolbar is provided to facilitate markup, including a ‘Link to Post’ button that pops up a new window containing a list of other posts. Selecting a post from this list incorporates a link in the current post. This action is particularly useful for showing relationships between posts, as discussed in section 3.2.

Users can also categorise posts by adding metadata based on key-value pairs. Each e-Notebook includes the ‘Section’ metadata key by default. Clicking the button to the right of the field presents existing values in a drop-down list: the value assigned to this key is shown at the foot of the published post. Users can include additional metadata by providing a value for an existing key or by adding a new key. Existing keys appear in a drop-down list, with a text entry box for the addition of values. Users complete and publish their post by clicking the ‘Submit’ button. They can edit posts after publication, but the system requires them to give a reason for that edit, prior to updating the post. Users can change metadata when editing an existing post.

The default ‘Section’ metadata key enables a top-level categorization of all posts. For example, in each of the bioscience e-Notebooks, Section keys were defined for the following post types: material, data, sample, procedure, safety, template, and note. The top level of organization thus relates to the role of the post, the type of research element that it represents.

The System Description (provided as File S1) contains a section that explains the principal LabTrove objects and their relationships with each other. Among these objects are data files, which users can attach to a post, as described in the Attaching files section.

### Linking other posts

Links between posts are fundamental to LabTrove as an ELN. Links allows users to follow trails from materials, via processes, to products. LabTrove maintains a bidirectional link system, so if a post about an experiment includes a link to a post representing a reactant material, the reactant post will show that it is ‘linked by’ the experiment post. This makes it easy to trace, for example, which experiments used which materials. If a problem is found with one of the materials, it is easy to find all the experiments that used the particular sample.

### Attaching files

**Figure 3:**
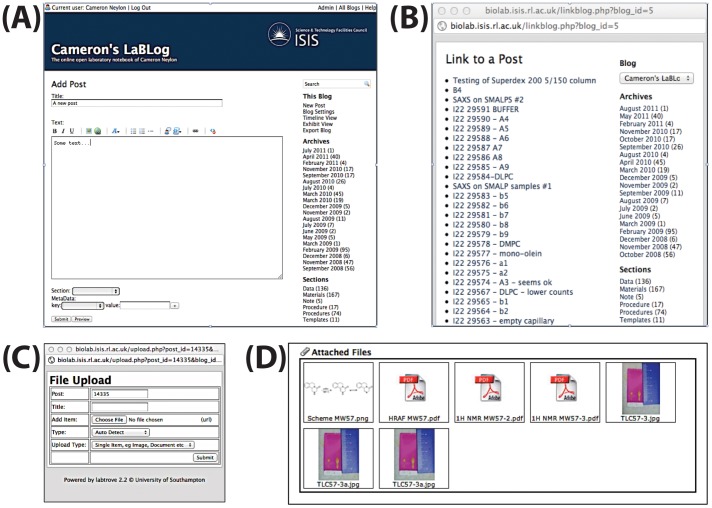
Tools used when adding and editing posts. (A) The editing view for creating text content, with a toolbar for markup, which includes an icon for linking to another post. (B) Clicking the link icon pops up a window from which an existing post can be selected to incorporate a link into the current post. (C) Data files can be added only after a post has been submitted. A popup window enables the selection and naming of the appropriate files, which are then attached to the post. (D) Thumbnails of the files can also be added into the text of the post.

A key feature of modern ELNs is the ability to link to files, which often contain the actual data generated by the experiment. With LabTrove, users can attach these files directly to an e-Notebook post that discusses the experiment or the data. Users can place in the body of the text a thumbnail representation of the file content. Clicking the thumbnail follows a link to the data file itself, allowing the content to be viewed or downloaded (see Figure 3). Typically for data generated by an instrument, we attach an image of the data for easy viewing and the raw data in a format appropriate for that instrument, such as a CSV, SPC (IR, Raman, NMR files, Galactic Industries 1997), JCAMP-DX files (http://www.jcamp-dx.org/).

### Interfacing with the LabTrove APIs

The APIs (application programming interfaces) allow other services to interact with the LabTrove system, which provides REST APIs to enable machine reading and posting of e-Notebook content, based on the export and import of single posts and collections of posts in XML format. LabTrove supports the writing and modification of content, the setting of key-value pairs, and the uploading and linking of data files. The LabTrove documentation includes a full description of using the REST API at http://www.labtrove.org/documentation/Using_the_Rest_API

### The one-item one-post system

We adopted this system during the first phase of development, owing to the need uniquely to identify each element of the research record, such as data, samples, and protocols. Specific posts create this identifier for many input materials, especially those bought in (e.g. *XhoI enzyme second batch*). We introduced posts of an equivalent nature for each product generated as part of an experiment.

This *one-item one-post* approach creates an implicit, yet simple, data model of the form: Object *has relationship to* Object. There are no explicit descriptions of what the relationships are, or of what any given Object represents. Metadata can be used to distinguish between general classes of item, such as material, procedure, or data file; and from the classes of items it is usually possible to extract an understanding of the relationship between two items. However, there is no explicit semantic content in this data model.

### Which research objects merit their own posts?

**Figure 4:**
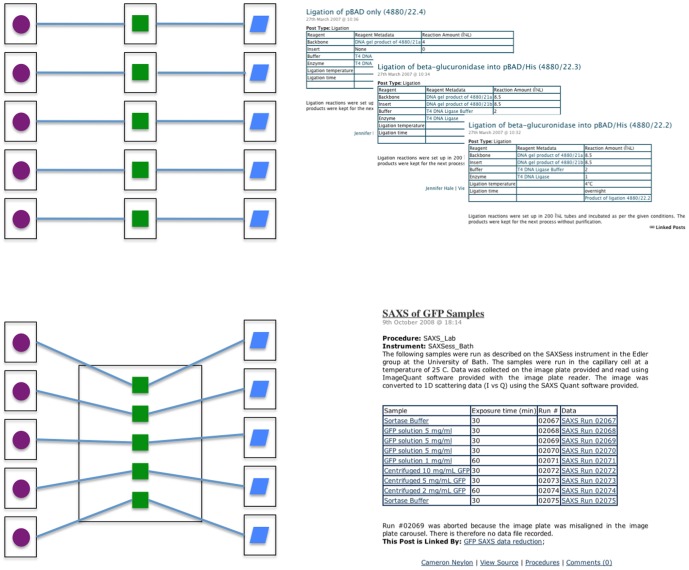
Diagram illustrating the alternative schemes for determining which research objects merit their own posts. Strictly, scheme (A) is more correct, as every individual process or reaction receives its own post and therefore has its own URI. This approach has the advantage of creating a discrete endpoint for every relationship between inputs, processes, and outputs. However, this scheme has the potential to create an unsustainable burden on the user and to render the records difficult to read as a laboratory notebook. In scheme (B), which we usually adopt, objects (in this case, samples and data) have their own posts but repeated processes are collected together and the relationship between specific inputs and outputs is recorded implicitly in a table. Posts that use this approach are more human-readable, but if necessary specific values can still be extracted programmatically into structured data.

For materials that would be stored or that might be used for a different purpose, we adopted the policy that each container should have its own post. Common molecular biology procedures, such as running a gel, purifying plasmid DNA, or PCR (polymerase chain reaction), also have their own post and their own set of outputs. The situation was less clear for individual procedures that were repeated, for example, parallel PCRs. Although the logical approach would be for every procedure to have its own post, the resulting structure would be almost impossible for humans to read. We therefore took the decision to keep sets of procedures together in one container post and to infer the relationship between inputs and outputs for a specific procedure from the organisation of the procedure post (see Figure 4).

This pragmatic approach generates an e-Notebook in which a human user can read the procedure posts in essentially the same way as in a traditional laboratory notebook. The associated posts, relating to the input materials and products, are used essentially as token and in most circumstances do not need to be viewed directly. Materials posts can, however, hold information that may be of use to other systems, or to the user, such as chemical identifiers, suppliers, physical properties, and safety or chemical incompatibility data.

### Advantages and implications of the one-item one-post system

The unique association between a post and a research element affords advantages for monitoring the use of materials and samples and for inspecting research processes. The LabTrove server generates a URI based on both the post identifier (as described in the System Description) and the address of the server. A URI resolver can therefore resolve any specific item in any LabTrove system worldwide and redirect the user to the appropriate post. The *one-item one-post* system also builds in a simple sample management and identification system. Research groups can use the unique URI to identify, track, and manage quantities of materials.

**Figure 5:**
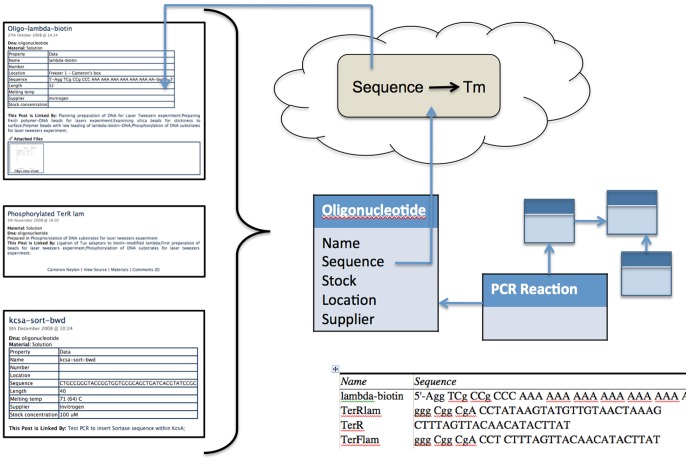
Extraction of unstructured and partially structured data from LabTrove posts. Similar posts, e.g. those that refer to a single type of material can be readily aggregated and parsed based on local knowledge of the structure. This information can then be tabulated or used to populate a relational database. It is also possible to extract parameters, such as the sequence of an oligonucleotide, do external analysis, and pass this information back into the post. See Table 1 for an example of structured data obtained from posts that contain tables and the details of the methodology.

If the metadata is well organised it becomes possible to use the system to manage multiple databases of laboratory materials and stocks. For example, if all posts referring to *oligonucleotides* are labelled with appropriate metadata and the formats of the posts are consistent, the set of all *oligonucleotides* can be extracted from the system along with their properties. These properties could be provided individually or in tabulated form, either of which can be imported by external database systems, a process that could easily be automated for regular data extraction. By creating an informal structure that suits the user, but enabling the *arbitrary* structuring of that data to populate formal systems for specific analyses, we can gain the best of both worlds (see Figure 5).

**Table 1:** Extraction of structured data from partially structured LabTrove posts.

*Name*	*Sequence*	*Length*	*Location*	*Melting temp*	*Stock concentration*	*Supplier*
lambda-biotin	5′-Agg TCg CCg CCC AAA AAA AAA AAA AAA AAA AA-biotin-3′	32	Freezer 1 - Cameron's box			Invitrogen
TerRlam	ggg Cgg CgA CCTATAAGTATGTTGTAACTAAAG	33	Freezer 1 - Cameron's box			Invitrogen
TerR	CTTTAGTTACAACATACTTAT	21	Freezer 1 - Cameron's box			Invitrogen
TerFlam	ggg Cgg CgA CCT CTTTAGTTACAACATACTTAT	33	Freezer 1 - Cameron's box			Invitrogen
TerF	ATAAGTATGTTGTAACTAAAG	21	Freezer 1 - Cameron's box			Invitrogen
kcsa-sort-bwd	CTGCCGGGTACCGGTGGTGCGCAGCTGATCACGTATCCGC	40		71 (64) C	100 µM	Invitrogen
kcsa-sort-fwd	CGCACCACCGGTACCGGCAGACCTGCGCCGCCTCAGCCAG	40		74 (64) C	100 µM	Invitrogen

All posts with the metadata key “DNA” and the metadata value “oligonucleotide” were extracted from the LabTrove blog at http://biolab.isis.rl.ac.uk/camerons_labblog. Despite inconsistency in the description of the oligonucleotides it is possible to obtain structured data from posts that could be used to populate other tables or a relational database as desired. The tables in each post were parsed and data extracted from them, based only on the structure from the table. The extracted data were then converted to a tabular form in html and reformatted into this table. Five rows were removed representing posts where oligonucleotides were described only in free text form. Two columns, one with no entries, and one headed Property with ‘Data’ in every row, resulting from the top row of tables, are not shown for clarity. The columns have been re-ordered. The code that generated the tabular data as well as the data it generated is provided in the Supplementary Information.

An important consequence of identifying a post as a representation of a specific item, especially for materials, is the need to be explicit about whether the item is a specific instance or a class of instances. For example, an item representing ‘NaCl’ may refer generically to the material NaCl, to a specific supplier-provided bottle of NaCl, or to a specific, weighed-out, sample of NaCl. The highly flexible metadata framework is capable of distinguishing between all of these cases if the user so chooses [27].

We have adopted the general convention that a post refers to a specific instance of an item. For a material, the item is a specific container containing that material, which can then be conveniently labelled. We apply this convention in a pragmatic way to materials for which it is important to distinguish between containers. For example, each tube of a restriction enzyme would warrant a distinct post, whereas each bottle of NaCl generally would not, as NaCl is obtained as a highly pure substance with very high reliability. It is therefore very unlikely that distinguishing between two bottles of material would be necessary. By contrast, restriction enzymes can lose activity gradually. Failed molecular biology procedures are often traced back to a specific tube of reagent that is no longer active.

The existence of a URI for each object also makes it straightforward to describe a multistep process by tracking through a series of procedures and linked products. Users can page through a process manually by clicking the links for each step. Users can also use the metadata to select a subset of posts to show different views, such as just those procedures that correspond most closely to a traditional paper notebook.

### Templates

#### Templates for repeated or parallel procedures

To reduce the burden of entering the same information repeatedly, LabTrove provides templates to enable rapid publishing of such procedures. For example, biochemical procedures tend to be stereotyped, with PCR, restriction digestions, and gel electrophoresis generally carried out many times in the same way. In many biochemical procedures, especially when a series of reactions are carried out in parallel, a table is the natural way to present the input materials and to identify the products of each reaction. Text-based web systems such as blogs and wikis work well for plain text with the occasional picture incorporated, but are very cumbersome when introducing tables. By automatically creating markup code and inserting standard text, templates can facilitate both table construction and the recording of repeated protocols. In addition, templates can present links in a way that enables the correct URIs for input materials and reaction products to be inserted easily.

For the provenance features of ELNs such as LabTrove to be effective, it is important to capture the appropriate metadata. Given the well-known difficulty with persuading users of any system to record metadata this can be a significant challenge. It is therefore important to automate, as far as possible, the collection and recording of metadata. Templates can assist users to insert the appropriate metadata into newly created posts, for example by presenting existing values. Templates can both exploit and reinforce structure, stimulating a “virtuous circle” that encourages users towards consistent structured recording of their research even though the system does not actively enforce any structure.

Bradley and Samuel experimented with a similar approach in their SMIRP (Standard Modular Integrated Research Protocols) project [28]. Their aim was to provide direct access to the information related to an experiment or procedure without resorting to, for example, a research publication, having observed that there is generally no standard mechanism to trace such data within an ELN. SMIRP adopted a “protocol-experiment-parameter” representation, with logging of metadata such as the user ID and a date-time stamp. The SMIRP representation also enabled selected steps in a procedure to be carried out in an automated manner.

#### The LabTrove template system

**Figure 6:**
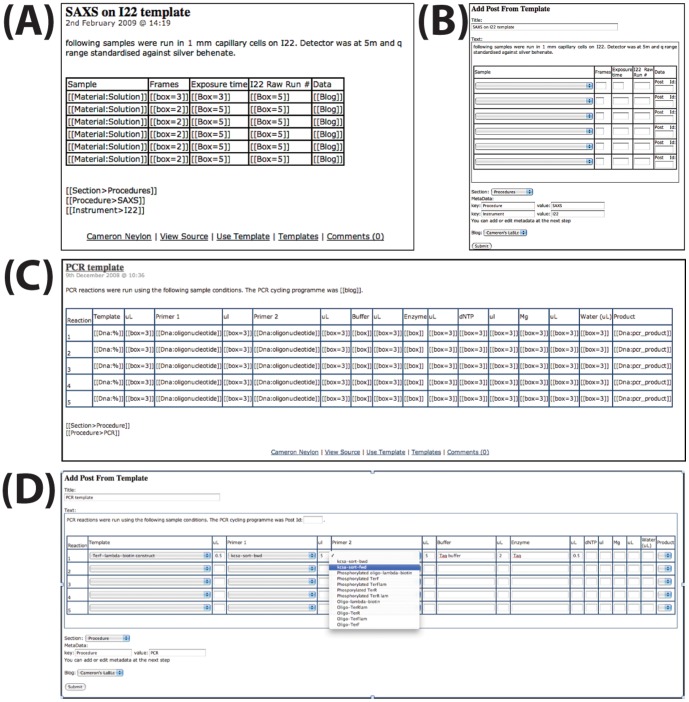
Extracts illustrating the design and use of templates. (A) A template post for a data collection type experiment in which a sample is measured with specific instrument parameters. The template includes placeholders for links to other posts in the ‘Sample’ column - [[Material:Solution]] – and in the ‘Data’ column. Note that the data itself would be created after the current template was used, so the post identifier data would be inserted during a subsequent edit. (B) The rendered template showing drop-down menus for selecting sample posts and text boxes for entering values. (C) A template for an experimental laboratory procedure involving a range of input samples and a single output product. (D) The rendered template being populated.

The template system enables users to design and deploy templates for creating new posts (see Figure 6). Each template is itself a post that contains, in addition to any normal text and markup, placeholders that LabTrove interprets when the template is rendered. Templates can present text boxes for entering data, which can include a link to a specific post, or a drop-down menu populated with the titles of relevant posts. Selecting a post from the drop-down menu creates a link to that object when the new post is published.

Placeholders for metadata are inserted into the template post in the form: [[Key>Value]], enabling users to associate specific metadata with the published post. By deploying templates that standardise the metadata for specific types of post, we can mitigate the issues related to consistency in metadata recording. The resulting, more consistent, structure makes the ELN system more powerful. Templates can include an arbitrary number of placeholders for metadata key-value pairs. Examples of LabTrove templates can be obtained from the myExperiment website by logging in, selecting the *Workflows* tab, and clicking the link for the *LabTrove Template* type. See for example the set of templates produced for the characterization work in a synthetic chemistry laboratory and shared via the MyExperiment [29] site (http://www.myexperiment.org/).

In summary, templates provide a rapid and easy way to generate posts with reproducible formatting and reduce the need for users to use the BBCode markup language, particularly for tables. Templates also enable appropriate metadata to be associated with posts. The key advantage of templates is that they encourage consistency and good record keeping. For example, templates cannot refer to input materials unless they already have been entered into the system. Templates automatically encourage users to provide the metadata that is often inconsistent or incomplete when entered manually.

### Curation and publication

Each post in a LabTrove e-Notebook has a URI, so a reference in a publication presents the e-Notebook as a form of repository ELN. Potentially, there are long term curation issues with this approach, so alternative ways to export the information and records to other repositories are required. Individual posts can be exported in XML format or converted to PDF, which could be routed to an industry standard curation system to protect intellectual property rights.

**Figure 7:**
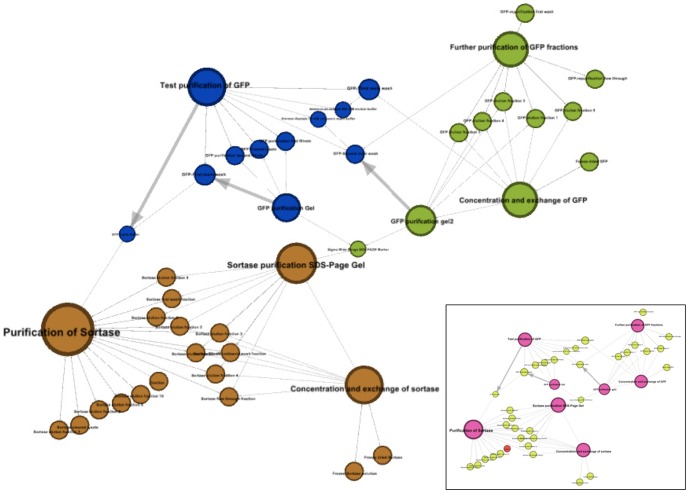
Visualization of posts as a network of resources. The full set of posts from a single e-Notebook was obtained as an XML document and parsed [30] into Geph (http://gephi.org/) graph format [31]. A subset of posts from a specific time frame, during which a number of protein purifications were being carried out, was selected and automatically grouped by sub-network modularity. The modularity analysis successfully differentiates the posts in three separate but parallel experiments carried out at the same time. The inset shows the same graph colour-coded by post type: pink nodes are procedures, yellow nodes are physical samples, and the red node is a dataset.

LabTrove can export an entire e-Notebook (or a subset if required) in HTML, RDF or XML comprising a description of each of the posts and the links between them. The static but hyperlinked web document can be used as supplementary material associated with a journal paper. The linking information affords an entirely new way of visualising a laboratory book as a network, which provides a visual representation of the flow of materials and data through procedures and analysis. This view can also identify work that is incomplete or areas where a notebook entry is unfinished: the presence of an isolated post without connections would be one example. Links to published papers, or external to datasets, can be incorporated into the graph (see Figure 7).

## Discussion

### User experience and usability

Key to the successful development of any electronic laboratory notebook or laboratory recording system is its usefulness in practise. We have been using our LabTrove system since 2007 and have deposited just over 8,000 posts and 4.6 GB in the chem.blogs.soton.ac.uk Troves, nearly 10 GB in the xray.phys.soton.ac.uk e-Notebook, and the newer ourexperiment.org sites already have 500 MB of data. Bearing in mind that the majority of the material is text, these numbers show the use we make of the system. LabTrove is in regular use by several of us as our primary laboratory record.. The combination of the *one-item one-post* system, consistent metadata, and full text search provides us with a much more effective record than a paper notebook.

The user interface has not been a priority for development and currently remains rather crude. Many aspects could be enhanced, by including more visual editing tools, improving the drag and drop functionality and offering a range of accessible visualisation tools. At one level such improvements could actually have a negative effect on the system's core strength, its simplicity, but it is clear that improvements will be required to encourage more widespread adoption.

In some cases recording laboratory work in LabTrove slightly increases the burden on the researcher. The additional effort is required because the framework enforces a higher standard of record keeping. For example, the effective use of templates requires that input materials and samples have previously been entered into the system. However, this rigour in turn automatically generates a catalogue of these materials, which is available to the rest of the research group. Group-based and shared materials catalogues are usually poorly kept, if kept at all. Similarly, tracking samples across a research group or collaborative programme is often challenging. Our system can provide this tracking automatically, but only if the standard of record keeping is high enough to ensure that the catalogue is complete. When templates are designed well and used regularly, the burden of recording reduces and the benefits are maximised and realised. To some extent, this facility overlaps with the role of Laboratory Information and Management Systems (LIMS), thus demonstrating the versatility of LabTrove.

### Identity and attribution

Identity and attribution are crucial to the integrity of the scientific record. To attribute posts, the LabTrove system requires authentication of identity either by password or through an external authority providing a service based, for example, on LDAP (http://tools.ietf.org/html/rfc4511) or OpenID (http://openid.net). While LDAP is appropriate for authentication within an institution, open Troves and those that entail integration with external services require services such as OpenID. Thus, myExperiment uses OpenID to authenticate identity, which was a major driver for using OpenID as an option for LabTrove.

Social issues can arise with regard to identity in the context of LabTrove. Recently, there has been significant discussion about the perceived threat to reasoned scientific discussion posed by anonymity and pseudonymity in the online world, with arguments generally supporting traditional notions of scientific or journalistic authority however Paul Raeburn, quoting Sharon Brownlee (Raeburn, P. The Atlantic on flu vaccines: Responses. Knight Science Journalism Tracker, http://ksj.mit.edu/tracker/2009/10/atlantic-flu-vaccines-responses), claims of authority based on a stable but pseudonymous identity [32] is equally acceptable, ultimately this may perhaps depend on legal interpretations of who you could sue!). More widely the idea of online identity is in flux with strongly held views on anonymity and pseudonymity not showing any immediate signs of resolution.

Within the context of reporting a record of science itself (as opposed to discussing its implications), the strong culture of attribution would suggest that complete anonymity is potentially damaging as it makes the attribution of ideas and results to a specific identifiable source difficult or impossible. Specifically it prevents the aggregation of attribution of a range of works to an identifiable party and makes it impossible for that party to build up authority. Anecdotally at least, allowing anonymous posting appears to correlate with antisocial behaviour, such as personal attacks and vandalism. We have never allowed anonymous posting

However there may be a role in science for pseudonymity, given that it would be appropriate where contributors, or a group of contributors, wish to maintain a single identity but not to allow that to be routinely associated with the a “real” identity [32]. In the case of LabTrove this can be supported via the use of OpenID functionality, providing a consistent identity, but not necessarily requiring credentials provided by a third party. In practice within the context of a research institution it is likely that existing identity infrastructure will be the most convenient to use, enabling integration with local sign on systems.

The more general issue of maintaining an authoritative and unique identity for each scientist is a significant one and well beyond the scope of this paper. Commercial efforts based on closed standards have begun to develop in this area as well as open standards such as Open ID initiatives and community efforts of which ORCID (http://about.orcid.org/) has gained the most attention [33]. The need for such a unique identifier for researchers is becoming more generally accepted and will be an important issue over the next several years. We would argue that any identity service for scientists must be based on open standards at a minimum and ideally should be not be under the control of a commercial provider. Whether this is feasible remains to be seen and the debate will no doubt continue for some time.

### Balancing structure and freedom - the metadata dilemma

Essentially, the LabTrove framework is semantically unaware; the system itself has no understanding of the content of the posts, the meaning behind their structure and connections, or the logic of the metadata. LabTrove offers the polar opposite of structured experimental descriptions and semantic tools such as the MyTea electronic laboratory notebook [7] [8] [15]. We have avoided experimental description formats such as FuGE (http://fuge.sourceforge.net/), or more widely the range of ontologies designed to describe biological systems and biological science [34].

Our experience is that freedom, and the ability arbitrarily to add or modify metadata keys and values, is critical to the recording of what is planned and what actually occurs in the small scale experimental laboratory. In this sense, we distinguish between recording a procedure, and describing an experiment. We believe that formal description frameworks like FuGE do not offer suitable models for the primary recording of experiments because they lack the flexibility to redefine the purpose of, rationale for, or conduct of, an experiment. For example, formal models are unable to handle an electrophoresis gel that contains samples from two or more unrelated experiments. Similarly, our experience is that the recording of our experimental work does not easily fit into the ISA-TAB model [35].

Much of this work is essentially exploratory, in some cases even playful, a mode of investigation that is very common in small-scale biological, chemical, and physical research. In particular where experimental approaches are being tested, the notion of what the investigation, or a particular assay is, changes over time. Samples can also be difficult to define clearly in some cases, particularly when their state changes over time in ways that are not yet known or understood.

However, the free form of our system carries the risk that its contents are not easily interpreted. If the metadata has no inherent semantics, has it any value? We have shown that if the metadata is used consistently, it is possible for the system to exploit this consistency in a useful and powerful way, by providing drop-down menus, regrouping items, and extracting data of specific types. The metadata is at least as useful to a human reader as the social tags common in Web 2.0 tools, as long as the values are clearly interpretable. The key question remaining is whether this free-form metadata could be mapped onto more structured controlled vocabularies.

We introduced the template functionality originally as a labour-saving feature, to enable the rapid generation of posts that were similar to previous posts. A key part of this functionality derived from the need for any material input to a procedure to have its own post, making it possible to generate a list of appropriate inputs based on the metadata associated with those materials. Templates afford ease of use only when users set metadata values correctly, which reinforces the need for consistent use of metadata. Thus, despite there being no vocabulary prescribed in advance, a consistent vocabulary can evolve of its own accord, and LabTrove reinforces the use of that vocabulary.

Consistent usage creates the opportunity to map the informal vocabulary that arises out of local practice onto externally constructed ontologies and controlled vocabularies (e.g. Ontology Listing. BioPortal, available from http://bioportal.bioontology.org/ontologies/). For example, the key-value pair DNA:oligonucleotide could be mapped onto the term ‘oligo’ from the Sequence Types and Features Ontology (SO:0000696) while DNA:pcr_product would map onto ‘pcr product’ (SO:0000006). In most cases, for simple materials, this mapping is straightforward. In many such cases it will be appropriate to prompt the user to use terms from existing controlled vocabularies by providing templates that use those terms.

Nevertheless, where controlled vocabularies or minimal information (MI) standards do exist [36], it will be valuable to use them as much as is feasible. We consider a good approach to be the automatic generation of templates from a combination of marked-up text and MI standards, which would simplify template creation, encourage consistency in recording, and improve the machine readability of the record.

For procedures the mapping is significantly more complex, owing in part to the inherent complexity involved in describing a protocol or procedure compared to the characteristics of a physical item. Further complexity arises from the wider range of ontologies, controlled vocabularies, and MI standards that are available, often developed for differing purposes and communities. For instance, our currently preferred approach for describing electrophoresis (Procedure_type:electrophoresis, Electrophoresis:agarose, Electrophoresis:SDS-PAGE) maps well onto the SepCV controlled vocabulary (HUPO Proteomics Standards Initiative, ontology entries, Available from: https://psidev.svn.sourceforge.net/svnroot/psidev/psi/sepcv/trunk/sep.obo and http://www.psidev.info/) eg. The mapping sep:00143, electrophoresis, sep:00171, agarose electrophoresis, sep:00173, sodium dodecyl sulfate polyacrylamide gel electrophoresis. However, the question arises whether the description should also adhere to the proposed Gel Markup Language, (http://www.psidev.info/index.php?q=node/69) which is intended for use in proteomics studies.

These formal approaches, while appropriate for high-throughput and data-intensive experiments, can be very burdensome for the simple generic experiments that occupy most time in the laboratory. For example, in a gel based assay, is a band present or absent? The additional benefits of a detailed and machine-readable description might not be worth the additional effort required to record all the required data or to build sufficiently generic tools for capturing the data at source.

### Balancing structure and freedom – practical experience

Overall our practical experience is that it is more effective to allow complete freedom in the recording process, if that recording process can be designed so as to encourage consistency of vocabulary and structure. The nature of the research process is sometimes haphazard, in that it is frequently difficult in advance to place useful bounds on where an experiment starts and stops, what its purpose is, or even what the input materials are (or are supposed to be). If there is internal consistency within the recording then it becomes feasible to apply the full power of structured descriptions such as FuGE and ISA-TAB for the purpose of specific communication.

Provided the metadata that is captured throughout the experiment is consistent and sufficiently fine-grained, it is feasible to map the local, free-form, metadata onto external controlled vocabularies, having chosen the appropriate description formats and standards. We have demonstrated this approach by constructing a relational database from posts describing oligonucleotides.

We recognise that in some circumstances, formal descriptions are suitable for directly recording the research process, and those descriptions can sometimes enhance the resulting record. However, for some forms of research, such formal requirements can *reduce* the quality of the record, either by relegating important observational or procedural elements of the record to second-class metadata elements or in some cases by discouraging effective use of the tools at all. In our experience, the choice of approach should be made on the basis of which structural models assist the user the most effectively both in creating a record of the research process that is as complete as is feasible, and in communicating the key elements of that process to other researchers.

### Linking the physical and digital worlds

LabTrove can generate both conventional barcodes and two-dimensional (QR) codes for each post. The combination of URI and barcodes provide both ready identification of specific items, and a simple sample management system and, with no additional effort. The ability to label everything that is entered into the system encourages users to ensure that new materials are indeed entered. In a multi-person laboratory the ability readily to identify who is responsible for a specific sample can be very useful. The ready identification of who has been using (and who has probably used the last of) specific reagents may also be conducive to improved laboratory harmony.

In a medium- to high-throughput procedure the use of templates along with a barcode reader can facilitate the rapid recording of relevant information. The template includes a placeholder that will accept a numeric value: the identifier of the post representing a specific sample supplied using a standard barcode reader.

### Enabling collaboration and communication

We have used LabTrove in a number of settings and found it to be very effective at enabling collaborative working. CN and JH were based at different sites, yet the ability to comment directly from a distance on each other's actions makes rapid communication and tracking of progress straightforward. We can track the posting of new experiments via a feed reader, and by using feed manipulators such as Yahoo! Pipes we can integrate a range of feeds, for example from the members of a research group, in different ways according to the needs of the consumer. We can pick up specific issues quickly and ideally solve them quickly too. Our experience is that many of the potential issues arising from group members being geographically dispersed simply vanish. The combination of tracking and commenting is essentially as effective for group members as being co-located, and can in some cases be more effective, given that a complete record is readily available and can be digested at convenient times. Moreover, our approach preserves a written record of interactions and comments, which can be useful in recording thought processes as well as attributing ideas.

The ability to define specific samples uniquely is particularly useful in cases where samples are shared or exchanged between groups. The *one-item one-post* system encourages the registration of individual samples, which makes complex collaborations more straightforward. Specific samples can readily be tracked through the system even when they may have passed through many hands. The ability to correlate a specific sample with detailed records of its preparation has great potential for making collaborations closer and more effective as well as rapidly identifying issues with communication. Similar reasoning applies to input materials, for which the *one-item one-post* system encourages the recording of specific batches, again making troubleshooting more straightforward, and enabling easy access to the batch-specific characterisation data provided by the manufacturer.

### Promoting open data and Open Notebook Science

Advances in organised collaborative networking afford the potential for sharing data in informal networks, with the wider community, or indeed with the public. There is a growing *open data* movement that aims to make the widest possible range of research data freely available. In conjunction, the agencies that fund research increasingly require that researchers make their data available openly to other members of the community.

While large and well-ordered datasets, such as the crystallography data for small molecules or proteins, can in most cases be made publicly available, publication presents a significant challenge for generic laboratory-based work. We built LabTrove for recording this raw data and its associated metadata, then making the record available by publishing it on the web. A blog-based research record is essentially web-native and thus affords more than just a digital repository. We can make the relevant data freely viewable and refer that data from a published paper. Some journals now allow authors to provide supplementary data in ‘mini-websites’.

The next step is *Open Notebook Science*, the description given by Jean-Claude Bradley to the practice of making the entire research record freely available as it happens. We have taken this step: we make the full record of the work in CN's group available as we capture it. It is the use of web-based recording systems in the laboratory that makes this practice, which is the logical extreme of openness in research, possible. A similar experiment in Open Notebook Science by Matthew Todd's group at the University of Sydney can be seen in the LabTrove http://www.ourexperiment.org/ sites, see for example to notebook pages,

- Pictet-Spengler route to Praziquantel (http://www.ourexperiment.org/racemic_pzq),
- Racemic Resolution of Praziquantel (http://www.ourexperiment.org/racres_pzq),
- Praziquanamine Racemization of PZQ and PZQamine (http://www.ourexperiment.org/rac_pza),
- Enantioselective Hydrogenation of dehydro-PZQ and derivatives (http://www.ourexperiment.org/enantio_hydgen),
- Synthesis of 3-substituted methylidene oxindoles (http://www.ourexperiment.org/synth_methyl_oxin).

It should be noted that such while such openness is supported and facilitated by LabTrove it is not a requirement. LabTrove will support private operation as well; whether single users and restricted groups.

Other initiatives that promote the open recording of science include: using a TeX-based document to generate a PDF that is made available on the web; providing code repositories for computational science projects; and a combination of free hosted services including wikis, blogs, and Google Docs. The OpenWetWare group (http://openwetware.org/) has developed wiki-based laboratory notebooks based on the MediaWiki platform, which are widely in the annual International Genetically Engineered Machines (iGEM) competition (http://www.igem.org/).

LabTrove is unique in providing a framework that generates a URI for each sample, procedure, and data file. Our system is therefore ideally positioned for the next stage in the development of Open Systems, in which both humans and machines will read and manipulate the research record. Each sample has an identity and can be tracked, enabling us to envisage an open access system in which samples can be requested by, and automatically dispatched to, outside laboratories. The system can record details of data analysis, which other workers can replicate using the raw data. The raw data can be reused in new ways to enable new analyses.

### Integrating with other tools

**Figure 8:**
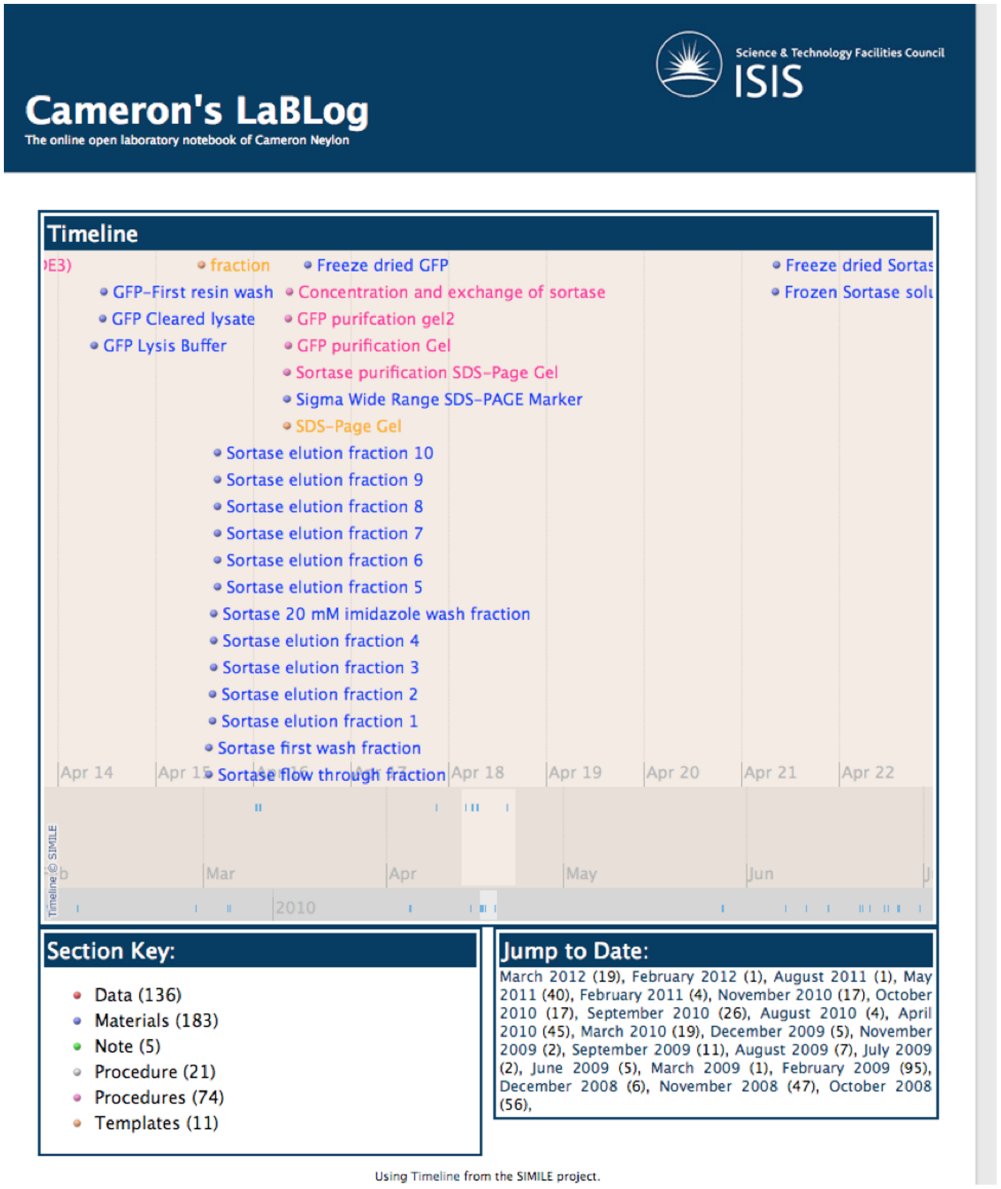
Example of a LabTrove TimeLine. Each item on the TimeLine is a hyperlink to the LabTrove entry.

As described in the System Description, LabTrove preserves all of its information in XML format, which facilitates integration with many other tools. In particular we have made considerable use of the SIMILE project from MIT (http://simile.mit.edu/), see Figure 8. We send to the SIMILE Timeline application an XML document containing the title, author, Section value, date, and URL of each post. The application displays a movable timeline showing when each post was added, colour coded by Section, with hyperlinks to each post. The result is a tool ideal for assessing what work has been done as well as an aid to finding older posts.

LabTrove templates in effect represent single steps in a workflow, connecting for example inputs and outputs via the process represented by the post. Further linking of posts provides a representation of a workflow. As such, they are potentially very useful objects to share between researchers working with similar procedures and protocols. We have implemented a link [37] with myExperiment [29] so that we can export the XML description, together with authorship details and a representative image of the template, from the LabTrove e-Notebook to myExperiment. From there, other users can be share the template or import it and use it in their own LabTrove notebook.

A key aspect of Web 2.0 websites is the ability to transfer, reuse, and ‘mashup’ multiple datasets. Although the application of these approaches to scientific datasets remains relatively limited, it is clear that the ability to integrate data using these tools has huge promise. We have used a range of freely available web-based tools to aggregate and reuse the data generated by the RSS feed from LabTrove. A simple form of mashup aggregates and filters the e-Notebook posts and comments. Yahoo! Pipes provide a simple user-configurable way of manipulating RSS feeds to generate a new user feed.

One of us (CN) has used Yahoo! Pipes to aggregate feeds from the ChemTools e-Notebooks, which can be found at http://tinyurl.com/63egpg, filter them to remove posts from CN, and then combine them into a single feed. CN views the output from the pipe in an RSS feed reader to track the work in his laboratory. The filter can also retain posts that contain certain keywords. Importantly, separate web services carry out this aggregation and integration. Where such services are well designed, it becomes straightforward to integrate a wide range of data sources without having to implement new function in LabTrove.

As discussed in section 4.3, we can also extract structured data from LabTrove to populate more sophisticated database systems, thus enabling more complex analyses. We can, in principle, rebuild these databases on demand, either for simple updates, or as the metadata framework evolves. However, all of these approaches to integration involve only reading outputs from LabTrove, rather than two-way interaction.

### Current deployments

We are currently involved in several collaborative projects that deploy LabTrove in a variety of scientific disciplines and will be the subject of future publications. It is perhaps a reflection of the researcher-centric nature of the LabTrove implementation that it has been deployed in a broad range of laboratory settings and for a variety of scientific applications. The case studies that we will be reporting in one of our future publications illustrate the use of a blog-based ELN in analytical, synthetic, and physical measurement laboratories. The applications we will describe include both one-off experiments and repetitive procedures.

## Future Developments

A stable environment is a prerequisite for all of the projects that will be the subject of a future publication, so our forward-looking plans are based on delivering LabTrove as a warranted service, with provisions for maintenance and long-term data storage. We can divide the main development requirements for the current system into two main categories: usability and integration.

Our issues with the user interface are very general concerns that apply across a wide range of Web 2.0 platforms. Our key issue, the straightforward preparation, generation, and (ideally) automated population of tables, is one that has not been solved well in any online platform. Meeting the general interface challenge of tables is well beyond the scope of what we, as a single group, can achieve. The broader issues of interface usability are relevant to any software development project and we are addressing them on an on going basis. Purely visual design issues are not currently a priority for us. However, it is not only visual interface issues that determine usability. We are aware of the need to extend the functionality provided by the LabTrove API, so are considering a range of enhancements.

Our most challenging area for development is integration with other services on a wider scale. In the preceding section, we discussed interchange with myExperiment but there is a clear need for much wider integration. Any electronic laboratory recording system should interact with the traditional published literature as well as with modern publication media such as wikis and blogs. Laboratory systems should also integrate with content-hosting sites, including scientific data repositories such as the PDB (Protein Data Bank [38]) and Genbank [39] and ideally with generic services such as Flickr, Slideshare (http://www.slideshare.net/), and YouTube (http://www.youtube.com/); as well as bookmarking and tagging services such as del.icio.us (http://delicious.com/) and citeulike (http://www.citeulike.org/). Moreover, if we are to realise the promise of open electronic record keeping, laboratory systems must interact with optimised and purpose-built search services.

At the import level, integration is a matter of preparing plugins or other mechanisms for embedding content from external services into LabTrove, for instance photos hosted on Flickr, or data in Google Spreadsheets (https://docs.google.com/), and presenting that content within the e-Notebook. At the export level, integration involves the automated deposition of appropriate data types with third party services acting as either backup or primary host. Many of these services have an application-programming interface (API) to support such automated deposition so the requirement is for us to provide facilities to export content that conforms to published schema. Some LabTrove users employ the API to capture data automatically from instruments and other recording devices. We intend to describe this technique, which is known as *auto-blogging*, in a future publication. However, our plans for future development are based on the presumption that facilities for embedding external content should be available both from the user interface and through calls to the LabTrove API. In the longer term, we hope that LabTrove will be recognised as one of a suite of tools for data annotation and sharing tools that enable reuse and facilitate reproducibility.

## Conclusions

The success of any electronic laboratory recording system depends on providing records that are sufficiently rich to allow the detailed reproduction or checking of any part of a reported process. LabTrove preserves the provenance of experimental procedures, analytical data, and materials usage by maintaining links between objects ranging from posts to physical artefacts. The provenance chain can be particularly important for sample management and tracking. Laboratory systems should also enable the reuse of data in new and unexpected ways, the efficient repurposing of materials, and the redeployment of experimental and analysis procedures for modified experiments. We have demonstrated how LabTrove templates facilitate not only the conduct of repeated procedures but also the exchange of established and proven techniques.

The scientific literature as it stands rarely, if ever, provides sufficient detail to enable other researchers to replicate the detail of a published study. Achieving the desired standard will require sophisticated recording systems that integrate human-generated journals with a wide range of instrumental and observational data, and are capable of presenting contents that are useful to, and readable by, both humans and machines. The LabTrove system we have presented (available from www.labtrove.org and SourceForge) provides a route towards reconciling the tensions and challenges that lie ahead in working towards these goals.

## Supporting Information

Figure S1 **The architecture and operation of the LabTrove system.**
(TIFF)

Figure S2 **The principal LabTrove objects.**
(TIFF)

Figure S3 **Schematic diagram illustrating the principal components of the PHP server process and the main flows of control between components.**
(TIFF)

Figure S4 **Schematic diagram illustrating the main database tables and their interconnections.**
(TIFF)

File S1 **System Description Appendix Document**. The LabTrove Electronic Laboratory Notebook: System Description.
(DOC)

File S2 **Supporting information notebook entry file.** An example of material extracted from a laboratory notebook. The notebook entries relevant to a specific published paper [40] were extracted and converted to a static html representation, which can be viewed in any web browser. The archive is made available via the figshare service [41].
(DOCX)

File S3 **Generating structured data from unstructured and partially structured LabTrove entries.** The zip file contains 3 files the python script (blogextract.py) used to generate the data described in Figure 5 and Table 1, the raw data generated as an html table (table.html), along with some minimal testing scripts (test.py). The blogextract.py script can be called directly from the command line to parse tables in any LabTrove blog, by providing the URL, along with the metadata key and values for the posts to parse. By default it will generate the same data as presented in table.html.
(ZIP)
