# Supplementary figures and images for: LabTrove: A Lightweight, Web Based, Laboratory “Blog” as a Route towards a Marked Up Record of Work in a Bioscience Research Laboratory
Source: PLoS One. 2013 Jul 23;8(7):e67460. doi: 10.1371/journal.pone.0067460 (PMC3720848; doi:10.1371/journal.pone.0067460)

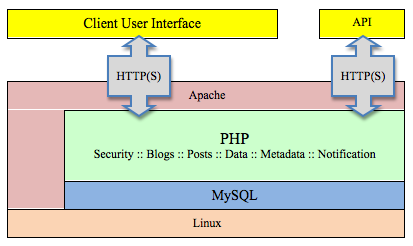

Supplement: Figure S1 — The architecture and operation of the LabTrove system. (TIFF) [file pone.0067460.s001.tiff]

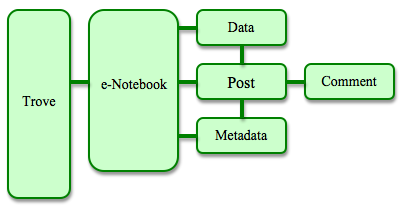

Supplement: Figure S2 — The principal LabTrove objects. (TIFF) [file pone.0067460.s002.tiff]

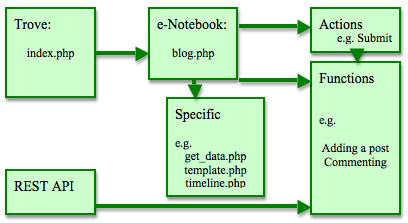

Supplement: Figure S3 — Schematic diagram illustrating the principal components of the PHP server process and the main flows of control between components. (TIFF) [file pone.0067460.s003.tiff]

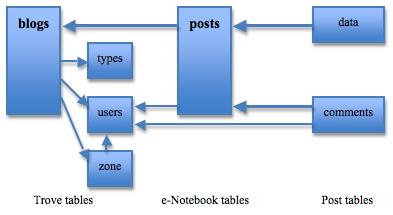

Supplement: Figure S4 — Schematic diagram illustrating the main database tables and their interconnections. (TIFF) [file pone.0067460.s004.tiff]
