# Supplementary material for: LabTrove: A Lightweight, Web Based, Laboratory “Blog” as a Route towards a Marked Up Record of Work in a Bioscience Research Laboratory
Source: PLoS One. 2013 Jul 23;8(7):e67460. doi: 10.1371/journal.pone.0067460 (PMC3720848; doi:10.1371/journal.pone.0067460)
Supplement: File S1 — System Description Appendix Document. The LabTrove Electronic Laboratory Notebook: System Description. (DOC) [file pone.0067460.s005.doc]

The LabTrove Electronic Laboratory Notebook: System Description

LabTrove is a blog-based system for recording laboratory processes and objects, as described in our paper, *LabTrove: A lightweight, web based, laboratory “blog” as a route towards a marked up record of work in a bioscience research laboratory*. This document describes the architecture of our implementation, the principal components of our design, and the primary aspects of the operation of the system. The main body of this paper describes the general functionality of LabTrove and how it is used in practice.

# Architecture

The LabTrove system employs a client-server architecture, with a PHP server running under Apache and a MySQL database. For our development system the Apache web server runs under Debian/Linux. This software combination is commonly known as LAMP.

Owing to the evolution of the LabTrove system, PHP script names and database table names do use the label *blog*, recognising that the technology that underpins a LabTrove e-Notebook is a blog.

Figure S1 illustrates the operation of the LabTrove system. Web clients issue POST requests using either the HTTP or HTTPS protocol to secure all traffic to and from the server. Apache passes the parameters from the calling URL string to the PHP script, which accesses the database and creates the HTML that Apache returns to the client. The REST APIs transmit requests in XML format.

# LabTrove objects

Figure S2 illustrates the principal LabTrove objects and their relationships with each other, summarised as follows:

- A *Trove* is a single LabTrove installation comprising any number of e-Notebooks grouped under one of three headings: Project Lab Books; Discussions; or Blogs. LabTrove manages access control to the e-Notebooks at the Trove level.
- An *e-Notebook* comprises any number of posts, any number of data files, and a collection of metadata keys that must include one or more values for the Section metadata key. LabTrove manages write access control to posts at the e-Notebook level.
- A *Post* can have any number of attached data files and any number of associated comments. Each *Post* must have a value for the Section metadata key and can be associated with any number of other metadata key-value pairs.
- *Data* comprises any number of data files, which are managed by the e-Notebook, and inherit the security and visibility settings of the particular e-Notebook. A data file can be attached to one post only and may be embedded by reference within that post. Optionally, the data file may be embedded within other posts.
- *Metadata* comprises any number of key-value pairs, of which only the Section key is mandatory for each post.

Strictly, links are not LabTrove objects, but are nevertheless fundamental to LabTrove as an ELN. Each link is a URI, which provides a unique form of identification for every element of the research process.

Each post consists of a title, an author, a date-and-time stamp, a body, and metadata. Each post also has an ‘Edit Reason’, which is set to ‘First Post’ when the author creates the post. The author supplies the ‘Edit Reason’ for any subsequent revision of the post. Each post has a unique numeric identifier that LabTrove supplies when the author creates the post. Each revision has a discrete identifier, which for the ‘First Post’ is the same as the post identifier. Each revision also has a discrete date-and-time stamp, but to ensure the correct sequencing of posts displays the date-and-time stamp of the ‘First Post’. All references to the post use the post identifier.

Each post can have any number of associated comments, which also have an author, a date-and-time stamp, a body, and an ‘Edit Reason’, but have no metadata.

The post body consists of free-form text that might include BBCode tags[[1]](#footnote-2) , for example to mark up lists, text highlighting, or images. The post body can also include links to other posts, using their post identifiers, and to external resources, using their URLs.

Any post with its Section key set to ‘Template’ can also include placeholder markup (although there is no requirement for such markup to be present).

When a user creates a post from a template, LabTrove interprets the placeholders and automatically inserts the appropriate markup with fields for entering text or numeric data, or values for metadata keys.

LabTrove provides post information to the client in formats other than HTML, those currently supported being XML, PNG, and JSON. Users wanting to view posts, comments, or revision lists in XML format, for example, modify the request URL in their client browser, replacing the ‘.html’ extension with ‘.xml’.

# Server process

Figure S3 illustrates the main control flows between the components of the PHP server process. The LabTrove download establishes a directory structure in which the ‘docs’ directory contains all the scripts that contribute to serving pages to the client. The other directories hold the scripts for all internal operations.

The index.php script serves the main page for a Trove and handles requests. When a user selects an e-Notebook, control passes to the blog.php script, which serves all pages relating to posts and other elements of the e-Notebook. Control can pass from the blog.php script in three circumstances:

- The user clicks a button that initiates processing within the e-Notebook, for example to submit a new post.
- The operation in the blog.php script requires interaction with the MySQL database, for example when adding a comment to a post.
- The operation in the blog.php script relates to a specific feature, for example when control passes to template.php to interpret placeholder markup.

When these operations are complete, control returns to the blog.php script.

The error codes that the server process returns are as defined by the HTTP protocol. For example, a generic processing error returns code 500, supplemented by a reason string. LabTrove does not maintain its own logs, relying instead on the Apache web server to track accesses and PHP errors.

The LabTrove architecture relies on plugins to provide features that are standard but system administrators might choose to customise. The Access control section of this document describes the authentication plugin.

*Historical note*: We based the first system - LaBLog - on a GPL blog called µBlog, which is no longer accessible but can be found via the web archive . However, we have now been through several development cycles and none of the original muBlog code remains, although we have retained the methodology of style templates and layout.

# Database

The MySQL database consists of twelve interconnected tables, some of which are for operational convenience. Figure S4 illustrates schematically how the main tables are connected.

In addition to administrative and descriptive information, the blogs table maintains a cache comprising the monthly archives for the e-Notebook, post references categorised by user, and a compilation of all the metadata associated with the posts in the e-Notebook. By using this cache LabTrove improves system performance.

LabTrove maintains metadata in the posts table, as a single field holding an XML string containing the metadata in the form of key-value pairs. The metadata field always contains at least one such pair, owing to the Section key being mandatory.

We considered the option of a discrete table holding the metadata in separate key and value fields, but decided in favour of the single field because it provides the flexibility to add new keys and values arbitrarily, without modifying the underlying database schema. Moreover, keys and values can both be changed at any time; keys can take any text value. However, this implementation does require the text string to be read with an XML parser to extract the key-value pairs. LabTrove optimises metadata filtering by updating the cache in the blogs table each time a user creates or modifies a post.

The posts table has a field that holds a list of any other posts that link to the post identified in that row of the table. When LabTrove saves a post, it checks the body of that post for references to other posts, using the link to add the source post to the ‘Linked to’ list for the referenced post.

Once entered into the database a record cannot be altered, thus ensuring that the provenance trail is complete, reliable, and reproducible. However, as described in section 2.1, ELNs do require a revision mechanism. When a user edits a post or a comment, LabTrove updates the relevant table, storing a new record, which includes fields holding the reason for the edit and the name of the user, and modifying the previous record to reference the new revision. The post and comment tables therefore contain a complete history and version control for each post or comment.

The current version of LabTrove stores data items as binary large objects in the MySQL database, subject to a threshold that is configurable by the system administrator. Above the threshold, LabTrove stores large data items in the file system. Storing all but very large files in the database has the advantage that a backup of the database contains all the Trove data; restoring that backup with a fresh copy of the code rebuilds the entire Trove. Future versions will include a user-configurable option to store data items separately. The current version does not provide selective backup, for example of a complete e-Notebook, although the Export option, as covered in the User Interface section, does serialise the e-Notebook in either XML or HTML format.

# User interface

Because the technology that underpins a LabTrove e-Notebook is a blog, the user interface format, as described in section 3.1, is standard for a blog. The content pane on the left displays either content in list form or a text box for editing an individual item, commonly a post. The content displayed can be at Trove, e-Notebook, or post level.

When a user creates a post, the editing view comprises a Title field, a Text field with a toolbar containing icons for introducing specific markup, and data entry field for the Section and other metadata key fields. When a user edits a post, the view includes additional fields for entering the reason for the edit and, if required, for attaching data files. When a user adds a comment to a post, the editing view includes only the Title and Text fields, with a toolbar for markup. When editing a comment, the view includes an additional field for entering the reason for the edit. The toolbar icon for linking to another post displays a popup window containing a list of posts to which the user is authorized to link.

For reasons of data integrity, the additional field for attaching data is not present in the editing view when a user creates a post. The editing interface does not create a record in the posts table until the user clicks the Submit button, whereas it does create a record in the data table at the time the user attaches a data file. If post creation does not complete, for example because the user discontinues preparing the post, the data file would be ‘orphaned’, in the sense that it would not be attached to a post. Attaching a data field during a subsequent revision ensures that the post does already exist.

Users can format the contents of the Text field with the BBCode markup language, either by marking up the text or by using the toolbar. However, the interface provides neither WYSIWYG (“what you see is what you get”) editing nor HTML markup. We chose BBCode in preference to HTML markup because the latter is more complex. It is our experience that users can readily describe how they want to format a post and not having a WYSIWYG editor offers no hindrance to users.

When a user views a post, with or without comments, LabTrove has rendered the BBCode as HTML, using the style sheets provided, for presentation in the client browser. Additional fields for adding metadata are present when a user creates or edits a post. Clicking the buttons to the right of the Section field and all other key fields provides a drop-down list of existing values and the option to add a new key. A Section value of ‘Templates’ marks the post as a template, causing the server process to interpret any placeholder markup and render the post for template-style input.

As with most blog interfaces, the navigation pane is to the right of the content pane. At the top, under the heading ‘This Blog’ are options pertaining to the e-Notebook and, when viewing or editing a post, the options pertaining to ‘This Post’. Below these options are the Archives for the e-Notebook and then the Sections, with each of the values assigned to that key appended with the number of instances. Similarly, below the Section list each of the other metadata keys is presented with the assigned values and number of instances. By selecting a Section or other metadata key, users can filter the posts listed in the content pane.

Under 'This Blog', there is also an Export Blog option; under 'This Post' the Export options enable users to serialize a post as XML and to capture an image of the post in PNG format. All views include a search field at the top of the navigation pane, which enables users to search the e-Notebook for occurrences of specific text strings. LabTrove displays the search results in the content as a list of posts, annotated with the author, date, and time of each post. LabTrove uses style sheets in CSS format to control the presentation in the client browser. Administrator users can modify these style sheets to customise the presentation format, for example to increase the otherwise fixed width of a template to accommodate wide tables.

# Access control: identity, authentication, and authorisation

In general, access to a Trove for any purpose other than browsing requires users to have a system account, for which each user must have an identity. LabTrove holds user identities in a database table: the fields include user name, e-mail address, and the authorisation level described later. For each e-Notebook, LabTrove also maintains a zone table, which holds a list of users who have specific authorisation level. In operation, the level of authorisation that a user has is the greater of the zone level and that user’s personal level.

The system administrator establishes the authentication method when setting up the LabTrove instance, so each Trove has a single method, determined by the plugin installed. For example, for users at the University of Southampton, the authentication plugin uses the University LDAP (Lightweight Directory Access Protocol) service. We have also implemented access and authentication via OpenID, which allows users to maintain a consistent identity over multiple systems. OpenID not only enables integration with third party services but also a federated model of LabTrove servers, in which the same user can have a single identity across multiple deployments.

LabTrove authorisation levels provide for a range of security and editing rights. In accord with the principles of open data, as discussed in section 4.6, the general policy is to make posts visible, but to require a system account for commenting, a to permit only the original author of a post to edit the body of the post. LabTrove defines authorisation levels according to the following layered model:

**View** - This is the default level, at which users can view posts but require authentication before adding a comment.

**User** – At this level, users can create posts and also their own e-Notebook. Note that LabTrove will check the identity of a user attempting to change an e-Notebook setting to ensure that the user is the owner.

**Editor** – At this level, users can read everything in the Trove, but can modify only their own posts or e-Notebooks.

**Admin** – At this level, users can edit anything, although every change is attributed by user name.

Only a system administrator can create a new LabTrove instance and set access privileges for that Trove.

1. http://www.bbcode.org/ [↑](#footnote-ref-2)
