# Supplementary material for: LabTrove: A Lightweight, Web Based, Laboratory “Blog” as a Route towards a Marked Up Record of Work in a Bioscience Research Laboratory
Source: PLoS One. 2013 Jul 23;8(7):e67460. doi: 10.1371/journal.pone.0067460 (PMC3720848; doi:10.1371/journal.pone.0067460)
Supplement: File S2 — Supporting information notebook entry file. An example of material extracted from a laboratory notebook. The notebook entries relevant to a specific published paper [40] were extracted and converted to a static html representation, which can be viewed in any web browser. The archive is made available via the figshare service [41]. (DOCX) [file pone.0067460.s006.docx]

**Laboratory Notebook Archive**

An example of material extracted from a laboratory notebook. The notebook entries relevant to a specific published paper [40] were extracted and converted to a static html representation, which can be viewed in any web browser. The archive is made available via the figshare service [41] <http://dx.doi.org/10.6084/m9.figshare.92444>.
