# Supplementary material for: LabTrove: A Lightweight, Web Based, Laboratory “Blog” as a Route towards a Marked Up Record of Work in a Bioscience Research Laboratory
Source: PLoS One. 2013 Jul 23;8(7):e67460. doi: 10.1371/journal.pone.0067460 (PMC3720848; doi:10.1371/journal.pone.0067460)
Supplement: File S3 — Generating structured data from unstructured and partially structured LabTrove entries. The zip file contains 3 files the python script (blogextract.py) used to generate the data described in Figure 5 and Table 1, the raw data generated as an html table (table.html), along with some minimal testing scripts (test.py). The blogextract.py script can be called directly from the command line to parse tables in any LabTrove blog, by providing the URL, along with the metadata key and values for the posts to parse. By default it will generate the same data as presented in table.html. (ZIP) [file pone.0067460.s007.zip › LabTroveExtract/table.html]

|  |  |  |  |  |  |  |  |  |
| --- | --- | --- | --- | --- | --- | --- | --- | --- |
| Length | Location | Melting temp | Name | Number | Property | Sequence | Stock concentration | Supplier |
|  |  |  |  |  |  |  |  |  |
|  |  |  |  |  |  |  |  |  |
|  |  |  |  |  |  |  |  |  |
|  |  |  |  |  |  |  |  |  |
|  |  |  |  |  |  |  |  |  |
| 32 | Freezer 1 - Cameron's box |  | lambda-biotin |  | Data | 5'-Agg TCg CCg CCC AAA AAA AAA AAA AAA AAA AA-biotin-3' |  | Invitrogen |
| 33 | Freezer 1 - Cameron's box |  | TerRlam |  | Data | ggg Cgg CgA CCTATAAGTATGTTGTAACTAAAG |  | Invitrogen |
| 21 | Freezer 1 - Cameron's box |  | TerR |  | Data | CTTTAGTTACAACATACTTAT |  | Invitrogen |
| 33 | Freezer 1 - Cameron's box |  | TerFlam |  | Data | ggg Cgg CgA CCT CTTTAGTTACAACATACTTAT |  | Invitrogen |
| 21 | Freezer 1 - Cameron's box |  | TerF |  | Data | ATAAGTATGTTGTAACTAAAG |  | Invitrogen |
| 40 |  | 71 (64) C | kcsa-sort-bwd |  | Data | CTGCCGGGTACCGGTGGTGCGCAGCTGATCACGTATCCGC | 100 uM | Invitrogen |
| 40 |  | 74 (64) C | kcsa-sort-fwd |  | Data | CGCACCACCGGTACCGGCAGACCTGCGCCGCCTCAGCCAG | 100 uM | Invitrogen |
